# On the Prevention of Insanity

**Published:** 1900-08-04

**Authors:** R. Percy Smith

**Affiliations:** Lecturer on Psychological Medicine, Charing Cross Hospital; president of the section.


### ON THE PREVENTION OF INSANITY.

Introductory Remarks delivered at the opening of the Section of Psychology by R. Percy Smith, M.D., F.R.C.P., Lecturer on Psychological Medicine, Charing Cross Hospital; president of the section.

In his opening remarks Dr. Percy Smith said that probably few circumstances connected with insanity had more stirred the public mind in the last few years than its apparent increase. Attempts had been made to show that this was indeed more apparent than real, that it was largely due to the accumulation of senile cases and to a diminished death-rate, but when all had been said there remained the undoubted fact that the numbers increased by leaps and bounds, that the authorities were constantly being confronted by the demand for more accommodation, and that the taxpayer had constantly to put his hand in his pocket to defray expenses. Turning, then, to the causes of insanity, he drew special attention to three of them, namely: insane inheritance, alcohol, and syphilis. We have not space, however, to do more than refer to his remarks on the first topic, which, so far as numbers are concerned, is clearly the most important of the three. There can be no doubt, he said, that if we are to hold our own in the struggle for existence with other nations it is of the highest importance that the race should not be largely reproduced by individuals whose nervous system is so unstable that it very easily gives way under those stresses which must, after all, occur with great frequency in the various conditions of life, but should be propagated mainly by those who are abundantly well equipped from the nervous point of view. It will be thought only a truism to say that if the race were only reproduced by those of stable nervous system and sound by inheritance, our existing asylums would soon be found to be larger than the requirements of the population. In the last half of the nineteenth century enormous strides have been made in the care and treatment of the insane. But I submit, he said, that with all this, which is most desirable, very scant attention has been given to the question how a great deal of insanity can be avoided, and to the fact that a great deal of insanity is preventable, and should be prevented. The Lunacy Law has been amended in the last few years so as to make it more difficult for those who urgently need care to be placed under care, while it has rendered it more difficult for patients to be detained and more easy to be discharged, as if the Legislature unconsciously arrived at the conclusion that by so doing it could diminish the numbers of the insane. Nevertheless, the numbers increase, and I submit that there is no more important problem for the twentieth century than the possibility of diminishing the numbers of the insane by their non-production. Among the more educated classes of the community there appears to be growing up some recognition of the importance of considering this question before marriage is entered upon, and it is often in the power of medical men to give advice which may at any rate prevent the union of two bad stocks. But love is proverbially blind, and among the unreflecting classes nothing is thought of marriage with a person of insane inheritance. Unfortunately, even medical men themselves are not always sufficiently alive to the dangers of propagating the race from defectively constituted individuals, and are sometimes apt to pooh-pooh any possibilities of risk in cases in which they are consulted, and to give the opinion that there is no danger in a marriage between two persons of neuropathic or insane stock, provided the individuals themselves have so far been apparently sound. It is, said Dr. Smith, very unlikely that the Legislature will ever intervene to interpose any legal bar to the marriage of persons of insane inheritance. There will continue to be "free trade" in marriage, and therefore it becomes all the more important to educate public opinion on the subject. It is safe to lay it down as a rule that no person of direct insane inheritance ought to marry with another of direct insane or neurotic inheritance, and that even an insane inheritance on one side only ought to be stated and made known beforehand. It is to be feared, however, that in some cases, where large property is concerned or where, perhaps, there are several marriageable daughters in a family of limited means, and where an avowal of insanity being in the family might "ruin their chances," concealment is apt to take place. When it becomes a question of advising as to the marriage of persons who have already suffered from a definite attack of insanity, marriage ought, as a rule, to be absolutely forbidden. Dr. Smith related a series of illustrations of the serious results which had followed from such marriages. A great difficulty is presented, he said, in the case of those who break down subsequent to marriage but recover, and where the woman is still of child-bearing age; and it is an important question when, for instance, a woman has had puerperal insanity after the birth of her first child, whether she ought further to reproduce her kind. One can but advise, the advice in most cases not being followed. Finally, it might be thought superfluous to comment on the iniquity of a congenital imbecile being allowed to marry were it not that several cases are recorded in which this has taken place, where large property has been concerned, and with, of course, the most deplorable results to the offspring. Even in the case of chronic epileptics, whether the disorder be of the nature of *petit mal* or *grand mal*, marriage ought not to be permitted.

